# The Prevalence of Elevated Blood Pressure and Its Association With Obesity in Children Aged 6-13 Years in Rural India: A Cross-Sectional Study

**DOI:** 10.7759/cureus.37916

**Published:** 2023-04-21

**Authors:** Bennie C James, Amrutha Venkateswaran, Agneeswaran A, Belgin Premkumar, Sanghavi B

**Affiliations:** 1 Paediatrics, Trichy Sri Ramaswamy Memorial (SRM) Medical College Hospital and Research Centre, Trichy, IND

**Keywords:** rural children, overweight, obesity, primary hypertension, elevated blood pressure

## Abstract

Introduction

Globally, hypertension is one of the major risk factors for cardiovascular disease. Childhood hypertension is one of the emerging conditions due to the increase in the prevalence of obesity in children in developing countries. An increase in blood pressure (BP) can be classified as secondary hypertension if it is caused by an underlying disease process or as primary hypertension if there is no identifiable cause. Primary hypertension during childhood often tracks into adulthood. The prevalence of primary hypertension, mostly in older school-aged children and adolescents, has increased in parallel with an obesity epidemic.

Materials and methods

This cross-sectional descriptive study was taken in different schools in the rural areas of Trichy District, Tamil Nadu, for a period of six months from July 2022 to December 2022; the study was done in children between six and 13 years. Anthropometry was taken, and blood pressure was measured using an appropriate-size BP cuff and standardized sphygmomanometer. Three values were taken at an interval for a minimum of five minutes, and the mean of the three values was calculated. Blood pressure percentiles were adopted from the American Academy of Pediatrics (AAP) 2017 guidelines for childhood hypertension.

Results

Out of 878 students, 49 (5.58%) students had abnormal BP, of which 28 (3.19%) students were categorized into elevated BP and 21 (2.39%) students had hypertension both in stages 1 and 2. Abnormal blood pressure was equally distributed in both males and females. More students were from the age group between 12 and 13 years (chi-square value: 58.469, P=0.001), which shows that as age increases, the prevalence of hypertension increases. The mean weight was around 31.97 kg, and the mean height was 135.34 cm. In this study, we found that 223 (25%) students were overweight and 53 (6.03%) students were obese. The prevalence of hypertension was 15.09% in the obese category and 1.35% in the overweight category (chi-square value: 83.712, P=0.000).

Conclusion

Due to limited data available on childhood hypertension based on the American Academy of Pediatrics (AAP) 2017 guidelines, this study highlights the AAP 2017 guidelines for early diagnosis of elevated BP and various stages of hypertension in children, and also, the need for early detection of obesity is essential for the implementation of a healthy lifestyle. This study helps create awareness among parents regarding the rise of obesity and hypertension in children in rural populations of India.

## Introduction

Globally, hypertension is one of the leading causes of cardiovascular diseases. The World Health Organization (WHO) estimates that around 40% of adults aged 25 years suffer from hypertension; the number of people with the condition rose from 600 million in 1980 to one billion in 2008 [1]. The prevalence was 5.4%-19.4% among children in the last two decades with an increase in trends [2-4]. Most of the studies done for hypertension in India have been conducted based on National Health and Nutrition Examination Survey (NHANES) guidelines, which show more prevalence of hypertension in children with obesity. These data were based on the 2004 Fourth Report on the Diagnosis, Evaluation, and Treatment of High Blood Pressure in Children and Adolescents [5]. The American Academy of Pediatrics (AAP) recommended newer guidelines in 2017 for new blood pressure (BP) levels to categorize elevated blood pressure and hypertension. According to the newer guidelines, blood pressure levels were lowered by 2-3 mmHg for children aged less than 12 years based on the percentiles for age, sex, and height [6].

The normal BP in adults is 120/80 mmHg (or lower). Elevated blood pressure is considered systolic of 120-129 mmHg and diastolic of <80 mmHg. Stage 1 hypertension is systolic blood pressure of 130-139 mmHg or diastolic blood pressure of 80-89 mmHg. Stage 2 is systolic blood pressure of ≥140 or diastolic blood pressure of ≥90 mmHg. This definition is based on potential since it relates the degree of BP elevation with a significant likelihood of subsequent cardiovascular events. Because hypertension-associated cardiovascular events (e.g., myocardial infarction (MI) and stroke) occur rarely in childhood, the definition of hypertension in children is statistical and based on the distribution of BP in healthy children, not outcomes. The clinical practice guideline on childhood hypertension, issued by the American Academy of Pediatrics (AAP) in 2017, maintains the same statistical approach to defining and categorizing childhood BP as in previous guidelines from the National High Blood Pressure Education Program (NHBPEP) [7,8]: normal BP: BP in the <90th percentile for age, sex, and height or <120/<80 mmHg (systolic/diastolic) for adolescents ≥ 13 years old; elevated BP: BP in the ≥90th percentile and <95th percentile for age, sex, and height or 120-129/<80 mmHg (systolic/diastolic) for adolescents ≥ 13 years old; and hypertension: BP in the >95th percentile for age, sex, and height or ≥130/80 mmHg (systolic/diastolic) for adolescents ≥ 13 years old. Hypertensive-level BP is further staged as follows: stage 1: between ≥95th percentile and <95th percentile + 12 mmHg or between 130/80 and 139/89 mmHg (whichever is lower) and stage 2: ≥95th percentile + 12 mmHg or ≥140/90 mmHg.

Previously, childhood obesity and being overweight were initially thought to be a disease in developed countries [9]. Overweight is defined as excess body weight relative to height, whereas obesity refers to surplus body fat [10]. In 1975, the prevalence of obesity and overweight among children and adolescents aged 5-19 was just 4% compared to 18% in 2016. The rise has been observed similarly among girls and boys. Now, more than 124 million children and adolescents (6% of girls and 8% of boys) were obese in 2016 [11]. There is a growing global health concern when it comes to overweight and obesity in children [12,13], and it poses a significant challenge to the healthcare system in developing countries [14]. Most of the studies state that the prevalence of obesity and overweight is more in developed countries than in developing countries [15]. However, the occurrence is increasing in developing countries in recent years [16].

## Materials and methods

Study design and setting

A cross-sectional descriptive study was carried out in different schools with the assent of parents in the rural areas of Trichy District, Tamil Nadu, India.

Study subjects 

The study was done on children between six and 13 years. Any child with a preexisting cause of hypertension (secondary hypertension) was excluded from the study. Before the study, permission was taken from the school, and an assent form was given to the parents.

Sampling method and sample size

Assuming a previous prevalence of 14.2%, a margin of error of 2.5%, and a confidence level of 95%, the calculated sample size was 830. A total of 878 children were included in our study, of which 463 (52.7%) were male and 415 (47.3%) were female.

Study period and study tool

The study was done for a period of six months from July 2022 to December 2022. Body mass index (BMI) can be calculated by dividing weight (in kilograms) by the square of height (in meters): BMI = weight (kg)/(height in m)^2^. BMI was plotted in the percentile chart for each child based on their age and sex using the Indian Academy of Pediatrics (IAP) BMI chart. If the BMI of the child is on the middle/orange line (23 adult equivalent lines) or between the middle orange line and the uppermost red line (27 adult equivalent lines) on the BMI chart, it means that the child is overweight. If the BMI of the child is on or above the uppermost red line (27 adult equivalent lines) on the BMI chart, it means that the child is obese.

Student anthropometry was taken, along with the height of the child, and BP was measured using an appropriate-size BP cuff, with an inflatable bladder width that is at least 40% of the arm circumference and bladder length that is 80%-100% of the arm circumference. Arm circumference is measured at a point midway between the acromion and the olecranon process. BP was measured using a standardized sphygmomanometer with an appropriate-size cuff covering two-thirds of the arm. BP was measured with the child in a sitting position, with the arm at the level of the heart, and after a five-minute rest. Three values were taken at an interval for a minimum of five minutes, and the mean of the three values was calculated. Blood pressure percentiles were derived from the blood pressure tables as given in the American Academy of Pediatrics guidelines 2017. The height that was measured during the study was plotted against the AAP percentile chart, and the BP percentile was measured.

Statistical analysis

Data will be entered in standard Microsoft (MS) Excel format (Microsoft Corp., Redmond, WA, USA) and will be analyzed using the Statistical Package for the Social Sciences (SPSS) (IBM SPSS Statistics, Armonk, NY, USA). In addition to the simple arithmetic calculations, the chi-square test was also used. For statistical significance, P<0.05 was considered statistically significant.

Ethical clearance

Ethical clearance was obtained from the Institutional Ethical Committee (IEC) of Trichy Sri Ramaswamy Memorial (SRM) Medical College Hospital and Research Centre (reference number 696/TSRMMCH&RC/ME-1/2022-IEC, number 142).

## Results

In our study, 878 students had participated from different schools in the Trichy District, and their BP were measured, along with their height and weight, with the arm at the level of the heart and after a five-minute rest.

Out of the 878 students, 463 (52.7%) were males and 415 (47.3%) were females. In this study, more students were from the age group between 12 and 13 years. The mean weight was around 31.97 kg, and the mean height was 135.34 cm (Table 1).

**Table 1:** Sociodemographic characteristics SD: standard deviation

Characteristics	Frequency (n=878)	Percentage
Gender		
Female	415	47.3
Male	463	52.7
Age		
6-7 years	89	10.1
7-8 years	88	10
8-9 years	101	11.5
9-10 years	90	10.3
10-11 years	87	9.9
11-12 years	113	12.9
12-13 years	310	35.3
	Mean	SD
Weight (kg)	31.976	10.5377
Height (cm)	135.349	15.2646

A total of 49 (5.58%) out of 878 students had abnormal BP, of which 28 (3.19%) students had elevated BP and 21 (2.39%) had hypertension both in stages 1 and 2. Elevated BP and hypertension were equally distributed in both males and females (Table 2).

**Table 2:** Distribution of BP in children with elevated BP and hypertension BP: blood pressure

BP (n=878)	BP range	Total number of students with abnormal BP (n=49)	Percentage
Elevated BP	90th-95th	28	3.19%
Stage 1	95th, 12 mmHg	19	2.16%
Stage 2	More than 95th, 12 mmHg	2	0.23%

**Figure 1:**
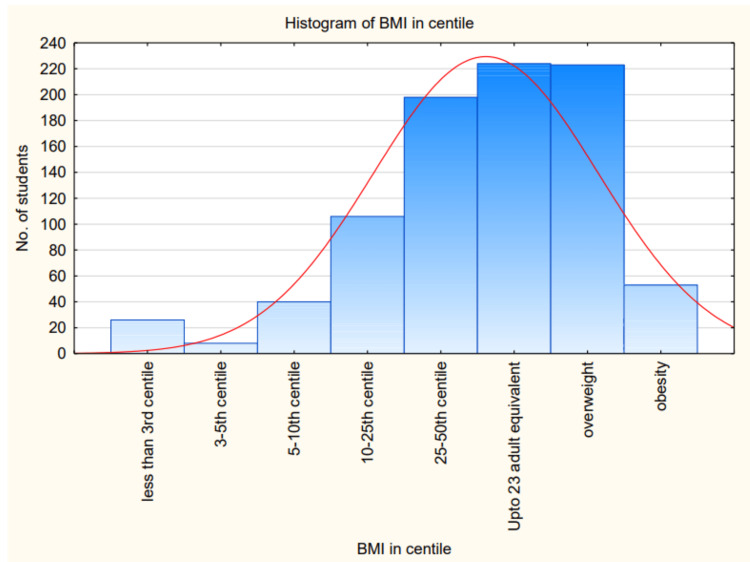
Histogram BMI: body mass index

Both their weight and height were taken from which their BMI was calculated and measured according to IAP BMI charts. In this study, we found that 223 (25%) students were overweight and 53 (6.03%) students were obese. When comparing the BMI of different students, the majority of them had BMI just below the overweight centile as shown in the histogram in Figure 1. This shows that although most of these children fall under the overweight group, these children have an increased risk of becoming obese in their adolescence.

**Figure 2:**
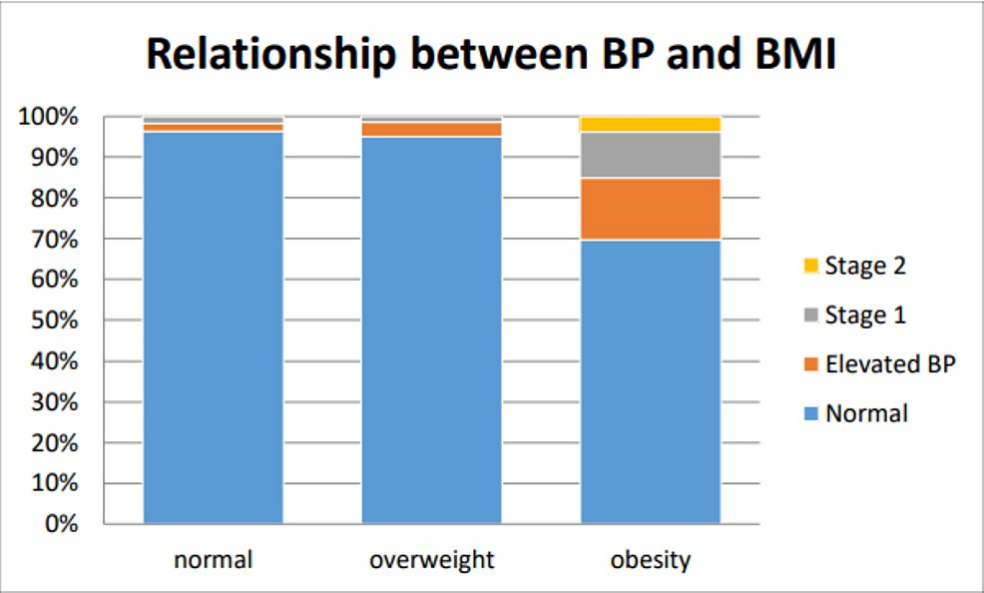
Relationship between BP and BMI according to 100% stack box BP: blood pressure, BMI: body mass index

The association between raised BMI and BP was correlated. There was an increased prevalence of hypertension in obese children as shown in Figure 2. The prevalence of hypertension was 15.09% in the obese category, while it was only 1.35% in the overweight category.

The majority of the students with normal BMI had normal BP (Table 3), but the majority of the students who had hypertension were seen in both overweight and obese groups (Tables 3-5).

**Table 3:** BP classification in children with normal BMI BP: blood pressure, BMI: body mass index

Normal BMI (n=602)	BP classification	Number of students	Percentage
Elevated BP	90th-95th	12	1.99%
Stage 1	95th, 12 mmHg	10	1.66%
Stage 2	More than 95th, 12 mmHg	0	0%
Normal	<90th percentile	580	96.35%

**Table 4:** BP classification in overweight children BP: blood pressure

Overweight (n=223)	BP classification	Number of students	Percentage
Elevated BP	90th-95th	8	3.59%
Stage 1	95th, 12 mmHg	3	1.35%
Stage 2	More than 95th, 12 mmHg	0	0%
Normal	<90th percentile	212	95.07%

**Table 5:** BP classification in obese children BP: blood pressure

Obese (n=53)	BP classification	Number of students	Percentage
Elevated BP	90th-95th	8	15.09%
Stage 1	95th, 12 mmHg	6	11.32%
Stage 2	More than 95th, 12 mmHg	2	3.77%
Normal	<90th percentile	37	69.81%

In our study, there was a significant correlation between BMI and BP in age groups 2, 5, 6, and 7 (Table 6).

**Table 6:** Association between BMI and systolic BP according to age P<0.05 is significant. BP: blood pressure, BMI: body mass index

Age group		Systolic BP	
		Chi-square value	P-value
Group 1	6-7 years	9.082	0.335
Group 2	7-8 years	31.177	0.008
Group 3	8-9 years	27.857	0.064
Group 4	9-10 years	10.570	0.061
Group 5	10-11 years	88.667	0.000
Group 6	11-12 years	77.078	0.000
Group 7	12-13 years	58.469	0.001

The association between BMI and hypertension was found to be statistically significant with a P value of >0.000 (Table 7). This study altogether shows that there is a significant increase in the prevalence of hypertension and obesity in children of advancing age.

**Table 7:** Association between BMI and systolic BP P<0.05 is significant. BP: blood pressure, BMI: body mass index

BMI category	Systolic BP category (%)				Chi-square value	P-value
	Stage 2 (n=2)	Stage 1 (n=19)	Elevated BP (n=28)	Normal (n=829)		
Obese (n=53)	3.8%	11.3%	15.1%	69.8%	83.712	0.000
Overweight (n=223)	0	1.3%	3.6%	95.1%		
Normal (n=576)	0	1.7%	1.9%	96.4%		
Underweight (n=26)	0	0	1%	96.2%		

## Discussion

The American Academy of Pediatrics has introduced newer guidelines that reduce the bias of higher BP in children compared to the NIH guidelines. This has reduced the prevalence of hypertension in children after the reduction of its threshold. Hypertension is one of the major causes of cardiovascular disease due to its relationship with obesity. A variety of studies have been done across the world, but not much has been done with the 2017 AAP guidelines. After searching PubMed for "prevalence," "hypertension," "AAP guidelines," and "India," only two journals conducted in India were found, which showed that the prevalence of elevated BP and hypertension was 15.9% and 35.1%, respectively, in children aged 10-12 years, and those children who are at higher risk of high BP were overweight and obese [17]. In our study, between six and 13 years of age, the prevalence was 5.58%, in which 3.19% had elevated BP, 2.16% had stage 1 hypertension, and 0.23% had stage 2 hypertension.

In another study conducted in Uttar Pradesh, where BP was measured in age groups of 10-19 years, it was found that the prevalence of hypertension increased as age advances [18]. Among 864 adolescents with hypertension, the prevalence was about 22.5% as per the AAP and 15.2% as per the NHBPEP. There was a high prevalence of hypertension in this study when compared to our study. The higher age group involved in this study could be the reason for the high prevalence. This study also suggested that there was a correlation between BMI and hypertension. A similar study done in the USA also had shown a high prevalence when comparing children from 2005-2008 and 2013-2016. As per the 2017 AAP guidelines, the prevalence was 5.7% in 2005-2008 and 3.5% in 2013-2016 [19], which was almost similar to our study.

In a previous study done in 2018 under 2004 NIH guidelines in India, in urban districts in Delhi, between five and 15 years of age, it was found that 3.1% of children fell under the prehypertension category. A family history of diabetes mellitus and obesity shows a significant association with childhood hypertension. In another similar study in West Bengal, based on 2004 NIH guidelines, between the age group of 6-18 years, the prevalence of prehypertension and hypertension was found to be 5% and 4.6%, respectively, and both were more common among children aged >15 years (10.3% and 15.5%, respectively). When compared to our study, the prevalence of hypertension is almost the same, but the guideline we followed was the AAP 2017 guidelines. All the above studies stated that hypertension was more common as age advanced, which was similar to our study.

Most of the studies in India were done on urban populations, which shows high prevalence, but it is not likely to say that rural populations are not affected. According to a systematic review and meta-analysis done in India, the prevalence of hypertension was slightly higher in urban children at 7% as compared to their rural counterparts at 5%. The study we conducted was completely done in rural areas, and the prevalence of hypertension in our study is 5.58%. Hypertension in this review has shown a continuously rising trend after the year 2005. Before 2005, the prevalence was only 3% and rose to 9% during 2006-2010, 7% in 2011-2015, and 6% in 2016-2020. We also observed that there is a significant correlation between obesity and being overweight, and hypertension, and there is a significant rise in the prevalence of hypertension over the last 15 years. According to this systematic review, about 10% prevalence was found in the states of Uttar Pradesh and Tamil Nadu [20].

Obesity is a major contributor to cardiovascular disease, metabolic syndrome, and hypertension, which is on the rise. In a systematic review, prevalence data from 28 states were collected, and it was found that obesity was more prevalent in the north than in the south side of India. In this review, data from around 52 studies conducted in 16 States in India were included in the analysis. The data collected after 2010 shows that the prevalence of childhood overweight and obesity was 19.3%, which was only reported at 16.3% in 2001-2005 [21]. One of the studies done in Chennai in 1981 and 1998 had shown a prevalence of obesity of 5.9% and 6.2%, respectively, in children aged 10-18 years [22]. Overweight and obesity were found to be 6.03% and 25%, respectively, in our study, which shows an increasing trend over the years.

There are several studies stating that there is a rise in hypertension and obesity in India. A study done in Delhi had shown that the prevalence of sustained hypertension among rural and urban areas was 5.7% and 8.4%, respectively [23]. The prevalence of obesity in rural and urban school children was 2.7% and 11%, respectively. In another study conducted in Odisha on the prevalence of hypertension and obesity, among 5,155 students, 10.4% were overweight and 3.6% were obese. A total of 190 (3.68%) students were found to have sustained hypertension. The number of female students with hypertension was more (4.47%) than the number of male students (3.2%) [24]. In both these studies, there was a significant association of hypertension in the obese group in comparison to the overweight and normal blood pressure group, which is similar to our study.

Limitations and strengths of the study

In this study, we only included children in the age group 6-13 years because as age advances, the prevalence of hypertension and obesity is more. Studies related to hypertension in children using AAP 2017 guidelines and in the rural population are fewer in India. We used the AAP classification in this study, and the study was conducted in a rural population. It shows that hypertension and obesity are emerging health problems in rural populations too.

## Conclusions

This study highlights the AAP 2017 guidelines for early diagnosis of hypertension in children. The need for early detection of hypertension and obesity is essential for the implementation of a healthy lifestyle and to encourage outdoor activity in children. This study helps create awareness among parents regarding the rise of obesity and hypertension in Indian rural children.
